# Self-Objectification and its Biological, Psychological and Social Predictors: A Cross-Cultural Study in Four European Countries and Iran

**DOI:** 10.5964/ejop.6075

**Published:** 2023-02-28

**Authors:** Silvia Gattino, Kamila Czepczor-Bernat, Angela Fedi, Anna Brytek-Matera, Mihaela Boza, Jérémy E. Lemoine, Reza N. Sahlan, Emma Wilson, Norma De Piccoli, Chiara Rollero

**Affiliations:** 1 Department of Psychology, University of Turin, Turin, Italy; 2 Institute of Psychology, University of Wrocław, Wroclaw, Poland; 3 Department of Psychology, Alexandru Ioan Cuza University of Iaşi, Iasi, Romania; 4 School of Psychology, University of East London, London, United Kingdom; 5 ESCP Business School, London, United Kingdom; 6 Department of Clinical Psychology, Iran University of Medical Sciences, Tehran, Iran; 7 ESRC Centre for Society and Mental Health, King’s College London, London, United Kingdom; 8 Health Service and Population Research, Institute of Psychiatry, Psychology and Neuroscience, King's College London, London, United Kingdom; Washington & Jefferson College, Washington, PA, USA

**Keywords:** self-objectification, body shame, body surveillance, cultural diversity

## Abstract

Although scholars started investigating self-objectification more than twenty years ago, only a few studies focused on men and even fewer have taken into account the cross-cultural dimension. Our study focused on the antecedents of self-objectification paying attention to the role of biological and sociodemographic variables (gender, BMI), psychological characteristics (self-esteem, perfectionism) together with social and cultural factors (internalization of media standards, influence of family and friends). Self-objectification was operationalized as Body Shame and Body Surveillance. A self-reported questionnaire was administered to 2165 adults living in four European countries (UK, Italy, Poland and Romania) and Iran. Ten regression models were performed (2 per country) to analyse the correlates of self-objectification. Overall, self-objectification emerged as a process affected by factors entrenched in psychological, biological, social and cultural domains, partially different for Body Shame and Body Surveillance. Findings showed the key role of self-esteem as a protective factor against Body Shame across countries. On the other hand, the internalization of media standards emerged as risk factor for both Body Shame and Body Surveillance in the five countries. Taken together, these results underline the complexity of self-objectification and the need to deepen research on this topic among non-Western countries.

Feminist analyses have provided a social constructionist account of the female body, arguing that in Western societies and mass media the female body is socially constructed as an object to be looked at and evaluated (Karsay et al., 2018; McKinley, 2011). Objectification Theory (Fredrickson & Roberts, 1997) states that women are often regarded as sexual objects by society, with the focus being placed on all or part of their bodies in a sexual context rather than on their abilities. When objectified, women are reduced to the status of “mere instruments” available for visual inspection, evaluation, and the pleasure of others (Bartky, 1990, p. 26). Through the pervasiveness of sexual objectification experiences, women are socialized to internalize an observers’ perspective upon their body. This process is called self-objectification and happens when women view and treat themselves as sexual objects to be considered and evaluated based on their appearance (Fredrickson & Roberts, 1997). Much of the literature considers self-objectification as being operationalized by two specific dimensions: body surveillance, the habitual monitoring of one’s appearance, and body shame, the result of falling short of internalized cultural standards of beauty (e.g., Grower & Ward, 2021; Karsay et al., 2018; Moradi & Varnes, 2017; Moya-Garófano et al., 2017; Zurbriggen et al., 2011).

One of the phenomena that plays an important role in contributing to this tendency is the internalization of women’s ideals of the body conveyed by the media (Vandenbosch & Eggermont, 2012; Vandenbosch & Eggermont, 2014) and the perception of the importance assigned by parents, friends and romantic partners to a woman’s physical appearance (Gattino et al., 2018; Midlarsky & Nitzburg, 2008; Ricciardelli & Mellor, 2012). The message that physical appearance constitutes the basis of identity, self-esteem, social and economic success and self-concept has been mainly directed at women (Unger, 1979). This external pressure strengthens women’s preoccupation with their bodies and causes body-related negative emotions, in particular shame and anxiety (Peat & Muehlenkamp, 2011; Rollero, 2013). As Calogero and Jost (2011) argue, the experience of self-objectification among women is also driven by broader ideological factors. In line with system justification theory (Jost & Banaji, 1994; Jost et al., 2004), these scholars emphasized that self-objectification can be seen as a consequence of the dominant sexist ideologies; justifying the gender status quo by directing women’s attention to managing appearance at the expense of other areas of life. This occurs even though gender disparity imposes significant costs on girls and women both as individuals and as a group.

Although self-objectification has been widely studied among women, recent research suggests that men’s bodies are also becoming objectified within Western cultures (Daniel et al., 2014; Davids et al., 2019; Moradi & Huang, 2008). Men seem to show lower levels of self-objectification than women (Calogero, 2009; Rollero & Tartaglia, 2016), however, compared to their older counterparts, young men pay more and more attention to their physical appearance (Rollero, 2013). This concern for one’s own image and attractiveness may reflect the intensification, in men, of a process of objectification that takes on characteristics different to female objectification, such as the tendency to consider muscularity more than thinness among socially shared standards of beauty (Rollero & Tartaglia, 2016).

Other biological characteristics relevant to self-objectification include age and Body Mass Index (BMI). Despite the growing demand for plastic surgery and “youth-restoring” cosmetics among older women, something which might suggest high feelings of self-objectification (Ring, 2000), some researchers in the field of objectification theory have found that as women get older, they feel less appearance-related social pressure and report lower levels of self-objectification (Greenleaf, 2005; Tiggemann & Lynch, 2001). In a study of European and American women aged 40–87 years old, Grippo and Hill (2008) found that although age did not moderate the association between self-objectification and body dissatisfaction, it moderated the association between habitual body monitoring and body dissatisfaction, so that this association was weaker for older women than for middle-aged women. BMI may also play a role in self-objectification by influencing one of its dimensions, body shame (Cella et al., 2020; Gattino et al., 2018). Several studies (Hunger et al., 2020; Meadows & Higgs, 2019; Pearl & Puhl, 2018) have found that internalized weight stigma and fat phobia resulting from experienced weight stigma play an important role in the relation between BMI and body shame. Research has also suggested that BMI itself can be considered a risk factor for body dissatisfaction and self-objectification in women (Fallon et al., 2014; Schaefer & Thompson, 2018). Indeed, previous research has shown that as BMI increases in women, feelings of body shame and self-objectification become stronger (Slevec & Tiggemann, 2011; Tiggemann & Lynch, 2001). The relation between BMI and body shame among women may be rooted in the negative association between BMI and thinness (World Health Organization, 2000). In contrast, the influence of BMI in men is not as linear, likely due to some men desiring a muscular body and other men desiring a lean body: as a result, both a low and high BMI may increase the risk of body dissatisfaction in men (Fallon et al., 2014; Gattino et al., 2018; Rollero & De Piccoli, 2015).

Research has also explored the relations between self-objectification and psychological variables, including self-esteem and perfectionism. There is strong support for the negative relation between self-objectification and self-esteem (Adams et al., 2017; Rollero et al., 2018; Tylka & Sabik, 2010). Perfectionism can be distinguished by two different dimensions: (1) self-perfectionism, an intra-personal dimension, is related to requiring perfection of oneself; it is demonstrated by strict standards of self-evaluation and a strong desire for success and the avoidance of failure; (2) social perfectionism, an interpersonal dimension, is associated with the belief that other people are critical of us and demand that we be perfect; it is manifested through the experience of external pressure to meet certain ideals and standards (Hewitt & Flett, 1991). In general, perfectionism can be adaptive when it serves to reach high personal standards, but it can also be maladaptive when it claims an excessive focus on the judgement of others and leads to excessive self-criticism (Frederick et al., 2016). In relation to self-objectification, Midlarsky and Nitzburg (2008) highlighted a role of perfectionism in predicting body dissatisfaction and disordered eating. Furthermore, perfectionism may promote the internalization of discontent with one’s body image in a more complex way, interacting with body shape and size, the effects of aging on appearance, and the influence of sociocultural pressures. Indeed, previous studies have found self-objectification to be associated with self-perfectionism and social perfectionism, both in women across cultures (Frederick et al., 2016; Gattino et al., 2018) and in men (for a review, Carrotte & Anderson, 2018). In sum, self-esteem and perfectionism can be explored alongside the psychological factors of self-objectification, as these relations are well established (e.g., Cella et al., 2020).

Self-objectification has been shown to be associated with social variables including the influence of parents, friends, romantic partners and the media (e.g., television, newspapers and magazines, social networking sites, websites). Studies carried out within the theoretical framework of the tripartite influence model have demonstrated the role that peers, parents and media play on body image (Keery et al., 2004). Both individual factors (emotional, cognitive, behavioural) and cultural factors (stereotypes, ideal body, body control, body importance) contribute to the development of body image (Sepúlveda & Calado, 2012). Based on these considerations, the importance that the immediate environment (parents, friends, romantic partners) assigns to physical appearance and the internalization of the ideals of beauty conveyed by the media affects how we perceive our own body, our emotions towards it and how we then behave (Myers & Crowther, 2007). A growing body of evidence has shown that the internalization of appearance ideals transmitted to us by our environment contributes towards self-objectification (Gattino et al., 2018; Vandenbosch & Eggermont, 2014). Through modelling behaviors and social pressure towards ideal body shape (thin for women, muscular and strong for men), family, friends, romantic partners and the media may affect body satisfaction, hence the degree of body surveillance and body shame (Katz-Wise et al., 2013; Kroon Van Diest & Perez, 2013). Regarding the role of the media, one cannot fail to notice that in recent years, access to mass media has been increasing. Whereas only TV and magazines once played a significant role, now the internet and social media are also of great importance in the globalization of beauty ideals (Feltman & Szymanski, 2018). Evaluating whether the media in culturally different countries plays a similarly important role in predicting self-objectification deserves further investigation.

Although the body of studies on self-objectification is now very large, most previous studies on this topic investigated a narrow sample of participants often from individualistic Western cultures or white college women from Anglophone nations, e.g., Australia, UK, and/or Western European countries, e.g., Belgium, Italy (Bernard et al., 2012; Loughnan et al., 2013; Vaes et al., 2011). Acknowledging this research may have captured only those forms of self-objectification specific to a given country, there was a need for research to include other nations. Therefore, research has recently been carried out in Asian (e.g., Japan, India and Pakistan; Loughnan et al., 2015) and Eastern European (e.g., Romania; Gattino et al., 2018) settings. Nonetheless, comparative studies are still scarce (Gattino et al., 2018; Gervais et al., 2015; Loughnan et al., 2015), particularly those involving both Central European countries and countries in the Middle East region. Referring to the statement that the body is a sociocultural construct, it should be remembered that culture may alter the intensity of self-objectification (Calogero, 2014; Fredrickson & Roberts, 1997). Therefore, in our study, we chose to examine factors organized into three blocks of content (biological characteristics, psychological variables, social variables) to detect risk and protective factors of self-objectification in culturally different countries, such as the UK, Italy, Poland, Romania, and Iran.

## The Cultural Context

Comparative cultural studies on self-objectification are strongly established in particular countries (e.g., Italy; Gattino et al., 2018), while others have not published any results yet (e.g., Poland). Cross-sectional studies in these countries might be of interest because of the historical, cultural, social and political differences between them. Besides the obvious differences between Western and non-Western countries, where the cultural, religious, and social norms governing the daily lives of men and women are very different, the European countries considered here also have some points of contact and customs that make it interesting to analyze and compare the risk and protective factors of self-objectification in these contexts. Both Romania and Poland were part of the former Soviet bloc and have undergone different processes of economic, political, and cultural development on their path of “westernization”. The effects of this process include increased exposure to Western views of body image, which is associated with increased dissatisfaction with one’s physique and an increase in eating disorders (Gattino et al., 2018; Rathner, 2001). Unlike Romania, however, Poland is a country in which the Catholic religion has always played a very important role. Even today, the Church represents not only a religious but also a cultural point of reference and plays an active role in the political life of the country, which it also strongly influences in social terms. Italy and the United Kingdom, on the other hand, are two Western European nations where body ideals—thinness for women and muscularity for men—are socially shared and ubiquitous. Nevertheless, there are some customs that differ between the countries (e.g., the role of religion, different family values, food as a cultural element and form of socialization) that make them socially and culturally not completely overlapping, and thus worth comparing.

Some specific contextual data for the five countries involved in the study are presented below.

### The United Kingdom (UK)

The UK is one of the most gender-egalitarian countries in the world and data from 2020 indicates that the UK ranks 21st out of the 153 countries (World Economic Forum, 2020). There is a certain amount of research on self-objectification conducted in the UK with results which are consistent with findings from other Westernized societies (e.g., Calogero, 2009; Fardouly et al., 2015; Ma & Loughnan, 2019). Both self-surveillance and body shame have been found to be positively associated with body guilt, media internalization, disordered eating and consideration for cosmetic surgery (Calogero & Pina, 2011; Calogero & Thompson, 2009a) and negatively associated with sexual self-esteem and sexual satisfaction (Calogero & Thompson, 2009b).

### Italy

According to the Global Gender Gap Report from the World Economic Forum (2020) Italy is one of the least egalitarian countries in Europe, ranking 76th out of 153 countries worldwide. Research on self-objectification is firmly established in Italy. It generally shares the findings from other Western countries and particularly emphasizes the role of the Italian mass media on objectification processes (Dakanalis et al., 2012; Rollero et al., 2018; Tartaglia & Rollero, 2015). Several studies conducted with Italian participants have shown that internalization of standards of beauty displayed in the media increases body surveillance and body shame, strongly predicts disordered eating behaviors, and reduces psychological well-being in women and men (Loughnan et al., 2015; Rollero, 2015; Rollero & De Piccoli, 2015).

### Poland

Poland is a country that is doing particularly well in terms of gender equality (it ranks 40th out of 153 countries; World Economic Forum, 2020) despite its recent history. Until 1989, Poland was part of the communist bloc and access to global media was significantly limited (Filas, 2009). However, since 1989, access to Western culture through the media has contributed to its Westernization (Filas, 2009), a pattern which has also been observed in other cultures (Becker et al., 2002). Although the reported prevalence of eating disorders and body dissatisfaction was previously very rare, exposure to Western standards of appearance and pressure from the mass media may lead to a change in ideals of beauty and body. Traditional values and beauty ideals are beginning to be less important than the ideals of attractiveness presented in the media (Becker et al., 2002). Similar changes began in Poland after 1989 (Filas, 2009; Pilecki et al., 2016), with research finding that Polish women now internalize those body ideals conveyed by the media almost as much as American women (Czepczor-Bernat et al., 2017).

### Romania

Romania ranks 55th in the EU in the Global Gender Gap Report (World Economic Forum, 2020). This situation may be partly explained by residual attitudes from its communist past (e.g., reproductive role of women, Andrei & Branda, 2015) but also the adoption of newer Westernized ways regarding gender relationships and consideration of women’s bodies (e.g., femininity is an ideal and a necessity; Svendsen, 1996).

These attitudes are still prevalent today due to the message shared by the media that individuals can control their appearance and should do whatever it takes to achieve perfection (Nanu et al., 2013, 2014).

Studies conducted among different age groups in Romania found that body self-esteem is positively correlated with overall self-esteem and body appreciation and negatively correlated with eating attitudes (Ivan & Daba-Buzoianu, 2016; Maguran, 2019). Additionally, the relation between body self-esteem and overall self-esteem was stronger for women, who were found to be more critical with their own body.

### Iran

Iran is considered as one of the least gender-egalitarian countries (World Economic Forum, 2020), ranking 148th out of 153 countries. This may be partly explained by the political situation in Iran in which the modest dress (i.e., hijab) has been legally mandated for women since the Iranian revolution in 1979. The adoption of a traditional Islamic dress style that minimizes public exposure may affect the degree to which Iranian women perceive pressure and internalization of a thin-ideal despite the increasing exposure to Western culture and media.

In countries where the hijab is voluntary (e.g. US, France, UK, Serbia), women who did not wear the hijab showed lower thin-ideal internalization, lower pressures for thinness and slightly lower disordered eating symptoms than Muslim women who did (Dunkel et al., 2010; Đurović et al., 2016; Kertechian & Swami, 2016; Swami et al., 2014). In Iran, where the hijab is compulsory, Iranian women endorsed a lower thin-ideal internalization, and lower pressures for thinness, than Western women (Schaefer et al., 2018). At first glance, these findings suggest that modest dress (e.g. wearing the hijab) may be protective against sociocultural influences; however, despite lower thin-ideal internalization and lower pressure for thinness, there is still an association with body dissatisfaction in women (Schaefer et al., 2018). Additionally, Pahlevan Sharif and colleagues (2019) found that the hijab does not decrease body image concerns in another community sample of women from Iran. Moreover, body image symptoms are prevalent in men, with few differences in body dissatisfaction across genders: specifically, Iranian men reported thin-ideal internalization, and pressures for thinness, which in turn, were associated with body dissatisfaction as well (Sahlan et al., 2020). Thus, Western-based sociocultural theories of eating pathology development, such as the Tripartite Model (e.g., Thompson et al., 1999) are likely salient to Iranian women and men (Sahlan et al., 2021).

### Aims and Hypothesis

This exploratory study builds on a previous research study that had investigated the antecedents of two dimensions of self-objectification among men and women in an Italian and Romanian sample (Gattino et al., 2018). Compared to that study, we added new cultural settings i.e., another Western country (United Kingdom), another Eastern European country (i.e. Poland) and a Middle Eastern country (i.e. Iran); the latter two very rarely considered in research on these topics. Secondly, we addressed the role played by additional psychological and social factors by including variables such as self-perfectionism and social perfectionism (among the psychological antecedents) and the specific influence of parents, friends and partners (among the psychosocial antecedents): this allowed us to unravel issues that had not been clarified in the previous work. This research, in particular, aims to identify risk and protective factors of self-objectification in five countries, which have historical, cultural, social and political differences.

Existing literature operationalizes self-objectification through the construct of objectified body consciousness, which refers to the degree to which people think about and treat their body as an object (McKinley, 2011). There are two main components of this construct that are usually measured (Calogero, 2009; Parent & Moradi, 2011; Tylka & Sabik, 2010): (a) body shame (BSH)—feeling shame when the body does not conform to cultural standards; (b) body surveillance (BS)—viewing the body as an outside observer.

Based on previous research, our hypotheses were as follows:

H1: the two dimensions of self-objectification are positively associated with sex and BMI—H1a and H1c—and negatively with age—H1b (Midlarsky & Nitzburg, 2008; Oksuz, 2008; Slevec & Tiggemann, 2011; Tiggemann & Lynch, 2001).

H2: BSH and BS are associated with psychological variables: (H2a) negatively with self-esteem (Tylka & Sabik, 2010) and (H2b) positively with personal and (H2c) social perfectionism (Midlarsky & Nitzburg, 2008).

H3: BSH and BS are positively associated with social variables, both those from the personal sphere (influence of parents—H3a; friends—H3b; partner—H3c) and those from the broader socio-cultural context (influence of media—H3d) (Green & Pritchard, 2003; Thompson & Stice, 2001; Vandenbosch & Eggermont, 2012).

To our knowledge, no study to date has examined these two dimensions of self-objectification by comparing countries in Western and Eastern Europe, and the Middle East, each with different socio-political and religious backgrounds. However, drawing on the literature (Adams et al., 2017; Karsay et al., 2018; Myers & Crowther, 2007; Tylka & Sabik, 2010), we expected that in all five countries both BSH and BS would be negatively associated with self-esteem and positively associated with media influence. Examining relations between the other variables across the countries was one of the aims of this study.

## Method

### Participants and Procedure

Of the 2566 heterosexual adults enrolled in the study, 2165 were valid cases (53.4% women) between 18 and 50 years of age (*M* = 33.5, *SD* = 9.0), residing in the UK, Italy, Poland, Romania and Iran (for more details, see Table a in the Supplementary Materials). Table 1 shows the demographic and physical characteristics of participants.

**Table 1:** Demographic and Physical Characteristics of Participants

Participant Characteristics				
Marital status				
Single	Married	Separated/Widowed		
49.2%	44.9%	5.9%		
Educational level				
College graduate	High school	Lower level		
28.1%	14.3%	57.6%		
Occupational status				
Employed	Student	Retired	Homemaker	Unemployed
72%	15.9%	0.3%	7.4%	4.3%
Physical characteristics^a^				
Normal weight	Overweight	Underweight		
63.4%	34.5%	2.0%		

^a^BMI ranged from 18.03 to 30.00 (*M* = 23.77; *SD* = 2.93).

The study protocol was approved by the Ethics Committee of the University of Turin. When available, we used validated scales in the language of each country. The scales which were not available in the target language were translated using the back-translation technique (Brislin, 1970). We recruited participants through snowball sampling and asked them to participate in a survey about personal and social issues. Every attempt was made to obtain a representative sample in terms of sex and age in each country. No identification data was collected to guarantee anonymity of the participants. The participants completed the questionnaire in approximately 20 minutes and received no compensation for their participation. The questionnaire was in paper format or online (Qualtrics platform) according to the site. To evaluate the factorial structure of the scales and to check for measurement invariance, we performed confirmatory factor analyses (CFAs) on the five samples and multi-group CFA. The analyses were carried out using the software AMOS 27. The results of these analyses are included as supplementary material.

### Measures

Self-objectification was measured through two sub-scales of the Objectified Body Consciousness Scale (OBCS; McKinley & Hyde, 1996): body shame (BSH; 8 items) and body surveillance (BS; 8 items). The body shame subscale evaluates the emotion individuals experience towards the perception that their physical appearance does not conform to the sociocultural standards of beauty (e.g., “When I can’t control my weight, I feel like something must be wrong with me”). The second detects the frequency with which respondents monitor their physical appearance (e.g., “During the day, I think about how I look many times”). Participants rate items on a 7-point scale ranging from 1 (strongly disagree) to 7 (strongly agree). Items have been reverse scored as needed, so that higher scores indicate higher levels of self-objectification.

Based on the results of the CFAs and multi-group CFAs, we deleted one item from each subscale due to low factor loadings: “I never worry that something is wrong with me when I am not exercising as much as I should” from the body shame subscale, and “I often worry about whether the clothes I am wearing make me look good” from the body surveillance subscale. Body shame showed satisfactory internal reliability in all samples (Cronbach’s α: UK = .85; Italy = .77; Poland = .81; Romania = .71; Iran = .83). The reliability of the body surveillance subscale was acceptable in the five countries (Cronbach’s α: UK = .83; Italy = .61; Poland = .77; Romania = .62; Iran = .82).

Perfectionism was assessed with two subscales of the Multidimensional Perfectionism Scale (MPS; Hewitt & Flett, 1991): Self-Oriented Prescribed Perfectionism (5 items; e.g., “One of my goals is to be perfect in everything I do”) and Socially Prescribed Perfectionism (5 items; e.g., “The people around me expect me to succeed at everything I do”). Both were rated on 7-point Likert-type scales, ranging from 1 (strongly disagree) to 7 (strongly agree). Higher scores indicate higher levels of perfectionism. Following the results of the CFAs and multi-group CFAs, we eliminated one item for each subscale due to low factor loadings: “I never aim for perfection in my work” from Self-Oriented Prescribed Perfectionism and “Those around me readily accept that I can make mistakes too” from Socially Prescribed Perfectionism. As found in other studies, (Bieling, Israeli, & Antony, 2004; Hewitt & Flett, 1991), reliability was acceptable for both subscales. Specifically, the internal reliability of the Self-Oriented Prescribed Perfectionism was acceptable in Italy (Cronbach’s α = .67) and satisfactory in the other countries (UK =.76; Poland = .75; Romania = .72; Iran = .80). The internal reliability of Socially Prescribed Perfectionism was acceptable in Romania (α = .63) and satisfactory in the other countries (UK = .77; Italy = .75; Poland = .71; Iran = .78).

Self-esteem was assessed with Rosenberg’s Self-Esteem Scale (RSES; Rosenberg, 1965). It includes 10 items (e.g., “I feel that I am a person of worth, at least on an equal plane with others”) rated on a 4-point scale ranging from 1 (strongly disagree) to 4 (strongly agree). Items were reversed when needed so that higher scores indicated higher levels of personal self-esteem. The scale showed good internal reliability in all samples (Cronbach’s α: UK = .92; Italy = .78; Poland = .81; Romania = .80; Iran = .81).

The Family and Friends Scale (20 items) was used to assess participants’ perceptions of the importance that their mother, father, friends and partner assign to physical appearance (Myers & Crowther, 2007; e.g. “My mother/ father/ friends/ partner encourage(s) me to care about my appearance in general”). Response options ranged from 1 (completely untrue) to 4 (completely true), with higher scores indicating greater levels of influence from parents, friends and partners. Given that in the five samples the two sub-scales concerning the influence of one’s mother and father showed a good internal coherence and a high correlation (.50 ≤ r ≤ .62), a single index related to the influence of both parents was calculated. As found in other studies (Myers & Crowther, 2007), the internal reliability (Cronbach’s α) of the scales was good: Influence of parents (UK = .85; Italy = .85; Poland = .84; Romania = .87; Iran = .87); Influence of friends (UK = 83; Italy = .81; Poland = .74; Romania = .81; Iran = .81); Influence of partners (UK = .87; Italy = .86; Poland = .79; Romania = .81; Iran = .82).

The internalization of the ideals of beauty conveyed by the media has been assessed through the Internalization-General Subscale of the Sociocultural Attitudes Towards Appearance Questionnaire-3 (SATAQ-3; Thompson et al., 2004)—10 items; e.g., “I compare my body to the bodies of TV personalities and movie stars”). Each of the 10 items was rated on a 5-point scale ranging from 1 (completely untrue) to 5 (completely true). Higher scores indicate higher levels of internalization of the beauty standards proposed by the media. In line with previous studies (Fitzsimmons-Craft et al., 2012; Myers & Crowther, 2007; Thompson et al., 2004), the internal reliability of the scale was very good in all groups (Cronbach’s α: UK = .96; Italy = .94; Poland = .96; Romania = .96; Iran = .96).

Data on sex, age, marital status, level of education and occupation were collected. To calculate BMI (Garrow & Webster, 1985) the participants had to indicate their weight and height.

### Data Analyses

First, we performed descriptive statistics, comparison of males and females and correlations between all the variables. Second, 10 hierarchical regressions were performed (2 per country using either BSH or BS as the outcome). The models included ten predictors organized into three blocks: biological characteristics (i.e., sex, age and BMI); psychological variables (i.e., self-esteem, self-perfectionism and social perfectionism); and social variables (i.e., influence of parents, friends, partners and the media). The three sets of variables were entered into the models following the above-mentioned order. These statistical analyses were carried out using IBM SPSS Statistics version 22.0 software.

## Results

### Preliminary Analyses

Analyses based on the overall sample indicated that males had lower levels of BSH and BS (BSH: *M* = 2.9, *SD* = 1.1; BS: *M* = 3.7; *SD* = 1.1) than females (BSH: *M* = 3.2, *SD* = 1.3; BS: *M* = 4.0; *SD* = 1.1). These differences are significant for both BSH, *t*(2153) = -5.82, *p* < .001, *d* = 0.25, and BS, *t*(2148) = -6.89, *p* < .001, *d* = 0.30, with small effect sizes (Cohen, 1992). At the country level, women had greater levels of BSH in the UK, Poland and Romania and they had greater levels of BS in all countries except Italy (see Table 2).

**Table 2:** Mean, Standard Deviation and Sex Comparison for BSH and BS Per Country

Five Countries' BSH and BS	Men *M* (*SD*)	Women *M* (*SD*)	*t*(*df*)	*d*
UK				
BSH	2.8 (1.1)	3.7 (1.1)	-2.44 (249)*	0.31
BS	3.1 (1.2)	4.2 (1.1)	-3.41 (249)***	0.43
Italy				
BSH	2.8 (1.1)	2.9 (1.2)	-1.09 (326)	0.12
BS	3.9 (1.0)	4.0 (1.1)	-0.57 (319)	0.06
Poland				
BSH	3.0 (1.3)	3.5 (1.4)	-4.75 (703)***	0.36
BS	3.8 (1.1)	4.4 (1.2)	-6.47 (703)***	0.49
Romania				
BSH	2.7 (1.1)	3.0 (1.1)	-2.87 (405)**	0.28
BS	3.6 (1.0)	3.9 (1.0)	-3.03 (407)**	0.30
Iran				
BSH	3.1 (1.0)	3.2 (1.2)	-0.88 (462)	0.08
BS	3.5 (1.1)	3.7 (1.1)	-2.14 (462)*	0.20

*Note*. *M* = mean; *SD* = standard deviation; *t* = t-test; *df* = degrees of freedom; *d* = Cohen’s d; BSH = Body Shame; BS = Body Surveillance.

*p < .05. **p < .01. ***p < .001.

Bivariate correlations among the five countries (for more details, see Table b in the Supplementary Materials) indicate that body shame and body surveillance were positively correlated in all groups.

### Regression Models

For all regression analyses, the assumptions of linearity, independence of errors, homoscedasticity and normality of residuals were examined and found to be satisfactory. We calculated the variance inflation factors (VIF). The values excluded multicollinearity among independent variables. Outliers were excluded and missing data were handled with listwise deletion. We performed the Bonferroni adjustment procedure leading to an alpha level of 0.01. Table 3 and Table 4 display the results of the hierarchical regressions using, (a) biological (i.e., sex, age and BMI) and, (b) psychological variables (i.e., self-esteem, self-perfectionism and social perfectionism) and social variables (influence of parents, partners, friends and the media) as predictors, and BSH and BS as dependent variables in the five countries. The biological characteristics were entered in Step 1, while the psychological variables and the social variables were entered in Step 2 and Step 3 respectively.

**Table 3:** Hierarchical Multiple Linear Regression Models Predicting Body Shame

	Step 1			Step 2			Step 3		
Predictors	Beta	*SE*	VIF	Beta	*SE*	VIF	Beta	*SE*	VIF
UK									
Female	.22**	.14	1.06	.19*	.12	1.07	.15*	.11	1.13
Age	-.24**	.01	1.03	-.16**	.01	1.09	-.08	.01	1.18
BMI	.36**	.02	1.08	.28*	.02	1.13	.24**	.02	1.15
Self-esteem				-.39**	.01	1.04	-.36**	.01	1.11
Self-perfectionism				.02	.07	1.19	.04	.06	1.22
Social perfectionism				.18*	.06	1.25	.10	.06	1.36
Influence of parents							.14*	.10	1.34
Influence of friends							.11	.11	1.85
Influence of partner							.12	.09	1.69
Mass media							.21**	.01	1.16
Adjusted R2	.17			.35			.49		
	*F*(3,243) = 17.51, p < .001			*F*(6,240) = 23.03, *p* < .001			*F*(10,236) = 24.19, *p* < .001		
Italy									
Female	.11	.15	1.24	.08	.13	1.25	.05	.12	1.29
Age	-.10	.01	1.14	-.08	.01	1.14	-.02	.01	1.21
BMI	.13	.03	1.33	.13	.03	1.37	.03	.02	1.46
Self-esteem				-.36**	.01	1.09	-.29**	.01	1.12
Self-perfectionism				.01	.06	1.41	-.01	.05	1.43
Social perfectionism				.25**	.06	1.43	.13*	.05	1.53
Influence of parents							.16*	.09	1.41
Influence of friends							.05	.10	1.75
Influence of partner							.21**	.08	1.61
Mass media							.28**	.06	1.21
Adjusted R2	.01			.22			.43		
	*F*(3,298) = 1.76, *p* = .16			*F*(6,295) = 14.75, *p* < .001			*F*(10,291) = 23.84, *p* < .001		
Poland									
Female	.24**	.11	1.16	.24**	.09	1.17	.17**	.08	1.22
Age	-.04	.01	1.06	-.05	.01	1.06	-.06	.00	1.07
BMI	.17**	.02	1.21	.16**	.02	1.22	.16**	.01	1.26
Self-esteem				-.30**	.01	1.11	-.22**	.01	1.18
Self-perfectionism				.13**	.04	1.28	.12**	.03	1.29
Social perfectionism				.26**	.04	1.37	.13**	.03	1.47
Influence of parents							.17**	.06	1.23
Influence of friends							.19**	.07	1.62
Influence of partner							.03	.06	1.53
Mass media							.30**	.03	1.33
Adjusted R2	.05			.28			.51		
	*F*(3,701) = 13.44, *p* < .001			*F*(6,698) = 47.53, *p* < .001			*F*(10,694) = 74.47, *p* < .001		
Romania									
Female	.23**	.11	1.15	.20**	.11	1.16	.14*	.10	1.30
Age	-.04	.01	1.08	-.03	.01	1.09	.05	.01	1.14
BMI	.17*	.02	1.24	.16**	.02	1.24	.10	.02	1.28
Self-esteem				-.32**	.01	1.02	-.35**	.01	1.06
Self-perfectionism				-.04	.06	1.43	-.04	.05	1.45
Social perfectionism				.14	.06	1.45	.07	.05	1.48
Influence of parents							.17*	.08	1.35
Influence of friends							.14	.09	2.04
Influence of partner							.05	.08	1.85
Mass media							.20**	.05	1.14
Adjusted R2	.05			.16			.30		
	*F*(3,377) = 6.98, *p* < .001			*F*(6,374) = 13.17, *p* < .001			*F*(10,370) = 17.60, *p* < .001		
Iran									
Female	.06	.11	1.00	.06	.11	1.05	.11	.02	1.21
Age	-.09	.01	1.16	-.06	.01	1.20	.02	.01	1.34
BMI	.16*	.02	1.16	.14*	.02	1.19	.10	.02	1.31
Self-esteem				-.28**	.01	1.07	-.20**	.01	1.13
Self-perfectionism				.01	.05	1.13	-.01	.05	1.16
Social perfectionism				.14	.04	1.26	.11	.04	1.30
Influence of parents							-.06	.10	1.41
Influence of friends							.17*	.09	1.80
Influence of partner							.02	.08	1.63
Mass media							.29**	.05	1.25
Adjusted R2	.02			.12			.22		
	*F*(3,382) = 3.55, *p* = .002			*F*(6,379) = 10.06, *p* < .001			*F*(10,375) = 12.15, *p* < .001		

*Note*. Beta = standardized coefficient; SE = standard error; VIF = variance inflation factors;

*p < .01. **p < .001

**Table 4:** Hierarchical Multiple Linear Regression Models Predicting Body Surveillance

	Step 1			Step 2			Step 3		
Predictors	Beta	*SE*	VIF	Beta	*SE*	VIF	Beta	*SE*	VIF
UK									
Female	.24**	.14	1.06	.24**	.14	1.07	.18*	.13	1.13
Age	-.17*	.01	1.03	-.15	.01	1.09	-.05	.01	1.18
BMI	.14	.02	1.08	.13	.02	1.13	.08	.02	1.15
Self-esteem				-.19*	.01	1.04	-.13	.01	1.11
Self-perfectionism				-.04	.07	1.19	-.04	.07	1.22
Social perfectionism				-.04	.07	1.25	-.04	-.07	1.36
Influence of parents							.01	.12	1.34
Influence of friends							.13	.13	1.85
Influence of partner							-.01	.11	1.70
Mass media							.35**	.06	1.16
Adjusted R2	.08			.10			.24		
	*F*(3,243) = 7.63, *p* < .001			*F*(6,240) = 5.77, *p* < .001			*F*(10,236) = 8.61, *p* < .001		
Italy									
Female	.01	.13	1.24	.01	.13	1.25	-.03	.12	1.29
Age	-.15	.01	1.15	-.15*	.01	1.15	-.08	.01	1.21
BMI	-.06	.03	1.33	-.05	.03	1.36	-.08	.02	1.43
Self-esteem				-.08	.01	1.08	-.04	.01	1.11
Self-perfectionism				.06	.06	1.39	.06	.06	1.42
Social perfectionism				-.01	.05	1.40	-.08	.05	1.50
Influence of parents							.08	.10	1.41
Influence of friends							.02	.11	1.73
Influence of partner							.00	.09	1.60
Mass media							.35**	.06	1.20
Adjusted R2	.02			.02			.14		
	*F*(3,293) = 3.15, *p* =.03			*F*(6,290) = 2.01, *p* = .06			*F*(10,286) = 5.73, *p* <.001		
Poland									
Female	.22**	.09	1.16	.23**	.08	1.17	.17**	.08	1.22
Age	-.18**	.06	1.06	-.18**	.00	1.06	-.19*	.00	1.07
BMI	-.06	.02	1.21	-.07	.02	1.22	-.05	.01	1.26
Self-esteem				-.12*	.01	1.11	-.04	.01	1.18
Self-perfectionism				.13*	.04	1.28	.12*	.04	1.29
Social perfectionism				.14*	.04	1.37	.05	.04	1.47
Influence of parents							.03	.06	1.23
Influence of friends							.07	.08	1.62
Influence of partner							.02	.06	1.53
Mass media							.35**	.04	1.33
Adjusted R2	.09			.16			.29		
	*F*(3,701) = 24.50, *p* < .001			*F*(6,698) = 23.20, *p* < .001			*F*(10,694) = 29.91, *p* < .001		
Romania									
Female	.13	.11	1.15	.13	.11	1.15	.10	.11	1.29
Age	-.11	.01	1.08	-.09	.01	1.09	-.05	.01	1.14
BMI	-.05	.02	1.23	-.05	.02	1.23	-.06	.02	1.27
Self-esteem				-.09	.01	1.02	-.08	.01	1.06
Self-perfectionism				-.01	.06	1.44	-.02	.06	1.46
Social perfectionism				-.10	.06	1.46	-.12	.06	1.48
Influence of parents							-.04	.09	1.35
Influence of friends							.03	.10	2.03
Influence of partner							-.00	.08	1.85
Mass media							.23**	.05	1.14
Adjusted R2	.03			.04			.08		
	*F*(3,377) = 4.92, *p* = .02			*F*(6,374) = 3.62, *p* = .02			*F*(10,370) = 4.30, *p* < .001		
Iran									
Female	.06	.11	1.00	.06	.11	1.05	.04	.12	1.21
Age	-.30**	.01	1.16	-.28***	.01	1.20	-.20**	.01	1.34
BMI	-.02	-.01	1.16	-.04	.02	1.19	-.05	.02	1.31
Self-esteem				-.19***	.01	1.07	-.15*	.01	1.13
Self-perfectionism				-.11*	.05	1.13	-.11	.05	1.16
Social perfectionism				.09	.04	1.26	.07	.04	1.30
Influence of parents							.11	.10	1.41
Influence of friends							-.03	.10	1.76
Influence of partner							-.02	.08	1.63
Mass media							.23**	.05	1.25
Adjusted R2	.10			.15			.20		
	*F*(3,382) = 14.65, *p* < .001			*F*(6,379) = 12.21, *p* < .001			*F*(10,375) = 10.80, *p* < .001		

*Note*. Beta = standardized coefficient; SE = standard error; VIF = variance inflation factors;

*p < .01. **p < .001

The increase in adjusted R2 indicated that the psychological variables contribute to the explanation of variation in terms of BSH above and beyond the biological characteristics: the adjusted R2 increased between Step 1 and Step 2 in the five countries. The adjusted R2 values showed a medium effect size in the Polish group and a small effect size in the other groups (Cohen, 1992). Additionally, the increase in adjusted R2 showed that the social variables contribute to the explanation of variation in terms of BSH above and beyond the biological characteristics and the psychological variables: the adjusted R2 increased between Step 2 and Step 3 in the five countries.

Moreover, results showed that sex and BMI were, among the biological variables, those most closely related to BSH at Step 3, although with some differences in the five groups. Specifically, a positive relation between being a female and high BSH (H1a) were found among British, Polish and Romanian participants, whereas a high BMI and higher levels of BSH (H1c) were only found among British and Polish participants, thus these hypotheses were partially supported.

Being of older age was negatively associated with BSH in the UK, Italy and in Poland, but the effects were slightly weak and not significant in every country, therefore Hypothesis 1b was not supported. Consistent with our hypothesis (H2a), self-esteem was negatively related to BSH across the five countries at Step 2 and 3. Among the psychological variables, it was the main predictor of BSH and, except the Polish and Iranian samples, for the other groups it was the variable most strongly associated with BSH. There was partial confirmation of the hypotheses for a positive association between the two forms of perfectionism and BSH. While self-perfectionism was positively associated with BSH (H2b) only in Poland, social perfectionism (H2c) showed a relation with BSH in Italy and in Poland. The influence of parents (H3a) was positively associated with BSH in all countries except in Iran, whereas the influence of friends (H3b) was positively related with the BSH only in Polish and Iranian groups where, among the social variables regarding the personal sphere, it was the one most strongly associated with BSH. Finally, the influence of a partner (H3c) was positively and significantly related to BSH only in the Italian group. In this sample, among the social variables related to the personal sphere, it was the most strongly associated with BSH. Thus, H3a, H3b and H3c were only partially supported. As expected (H3d), across the five countries, the influence of the media was positively related to BSH and in both the Polish and Iranian groups was the variable most strongly associated with BSH.

The increase in adjusted R2 indicated that the psychological variables contribute to the explanation of variation in terms of BS above and beyond the biological characteristics: except for the Italian group, adjusted R2 increased between Step 1 and Step 2. Additionally, results showed that the social variables contribute to the explanation of variation in terms of BS above and beyond the biological characteristics and psychological variables: the adjusted R2 increased between Step 2 and Step 3 in the five countries. However, the effect size is small in all samples (.08 ≤ Adj R2 ≤ .29).

The association of biological variables with BS differed between countries. At Step 3, sex was significantly related to BS in the UK and Poland (females had higher levels of BS), age showed a significant negative relation with BS in Poland and Iran. In Iran, moreover, age was the biological variable most strongly associated with BS. In contrast, no association between BS and BMI emerged in any country. Thus, hypotheses H1a and H1b were partially supported, while hypothesis H1c was not.

Similarly, there were cross-cultural differences in terms of psychological variables associated with BS. At Step 3, self-esteem (H2a) was negatively related to BS only in Iran, self-perfectionism (H2b) was significantly associated to BS only in Poland, while the association between social perfectionism and BS (H2c) was not significant in every country. Therefore, Hypothesis 2a and 2b were partially supported and Hypothesis 2c was not supported. Contrary to our hypotheses, the influence of parents (H3a), of friends (H3b) and of partners (H3c) were not associated with BS in any of the five countries. Consistent with our hypotheses (H3d), across the five countries, the variable most strongly associated with BS was the influence of the media (i.e., participants who internalized the ideals of beauty conveyed by the media).

## Discussion

This study aimed to investigate the antecedents of self-objectification, measured as body shame and body surveillance, in men and women aged 18 to 50 years, living in five culturally different countries (UK, Italy, Poland, Romania and Iran). Although scholars started investigating self-objectification more than twenty years ago, there are only a few studies among men and even fewer which have considered the cross-cultural dimension (Gattino et al., 2018; Loughnan et al., 2015; Trekels et al., 2018).

We found that both BSH and BS were higher for women than for men in the UK, Polish and Romanian samples, while Iranian women only had higher levels of BS but not BSH and there was no difference between men and women in the Italian sample. These findings are partly consistent with previous data indicating a greater vulnerability in women to self-objectification (Calogero, 2009; Grabe & Jackson, 2009). The absence of differences between women and men in our Italian sample is in line with a study indicating that the phenomenon of self-objectification is now transversal to both genders in Italy (Rollero & Tartaglia, 2016), as there is accumulating evidence that men and women are similarly overwhelmed by unrealistic body shape ideals and objectified in Italian media (see Dakanalis & Riva, 2013). This finding highlights how self-objectification is a socially constructed concept shaped by the social environment. Thus, cultural context and cultural differences should be considered when comparing and interpreting levels of self-objectification between men and women (Moradi, 2010; Trekels et al., 2018).

Regarding biological characteristics, we found that BMI was positively associated with BSH in the UK, Poland and Romania. However, BMI was not associated with BS in any of the five countries. This was only partially consistent with previous studies which found a positive relation between BMI and both BS and BSH (Ålgars et al., 2009; Slevec & Tiggemann, 2011). The lack of a relation between BMI and self-objectification is not new in the literature (Byely et al., 2000; Stice & Bearman, 2001). However, another possible explanation for these findings could be that we did not account for other variables that mediate the relations between BMI and BSH/BS, such as weight stigma and fat phobia (Hunger et al., 2020; Meadows & Higgs, 2019; Pearl & Puhl, 2018). Further studies would be needed to clarify this point.

Although we did not find a significant effect of age in the five countries, our results suggest that age may be a protective factor against BS. Indeed, older participants from Iran had lower levels of BS. No other significant effect was found in the other countries. This finding may be related to the different perception of ageing in this country, where—as in Asian cultures—old age is revered (Löckenhoff et al., 2009). Further investigations are necessary to understand whether the protective effect of age on self-objectification, found in some cultures but not others, is mediated by perceived ageism. Finally, being a woman was found to be a risk factor with respect to BS in the UK and Poland, and for BSH in the UK, Poland and Romania. There was no significant effect of sex in Italy and Iran. This latter result could be explained by different reasons. In Italy it could be interpreted as evidence of the pervasiveness of the phenomenon, which has become transversal to both females and males (Rollero & Tartaglia, 2016). In Iran it could instead be linked to the tendency of women to hide their bodies, an element that could reduce their sensitivity to self-objectification, at least in reference to their physical appearance (Dunkel et al., 2010; Kertechian & Swami, 2016).

Some interesting cross-cultural similarities have also emerged about the hypothesized relations between the psychological and social dimensions and self-objectification. On the one hand, self-esteem was negatively associated with BSH in the five countries and with BS only in Iran. This shows that self-esteem could be considered as a relevant protective factor against self-objectification. On the other hand, the internalization of media standards was positively associated with both BSH and BS in the five countries. This shows that the internalization of media standards could be considered as a powerful risk factor of self-objectification, irrespective of culture. These two variables play an important role on self-objectification in the five groups involved in the study and are more important than other variables traditionally considered, such as sex, age and BMI. The connection between self-esteem and body shame is a matter of debate (Choma et al., 2010; Mercurio & Landry, 2008). Tylka and Sabik (2010) suggest that individuals with low self-esteem may turn to the societal standards for guidance in determining their self-esteem. In addition, people with high self-esteem may be more inclined to accept their appearance as they, generally, feel satisfied with their other qualities. Our results seem to go in this direction and the different cultural contexts of participants underlines the importance of this psychological dimension.

Cross-cultural similarities regarding the media’s role as a risk factor for both BSH and BS are in line with previous literature, which suggests that people from different cultures may be exposed to the same content (Karsay et al., 2018; Tan et al., 2013; Trekels et al., 2018), including media, and probably share a set of common values because of global television and the internet (Craig & Douglas, 2006).

Conversely, findings about social and personal perfectionism were not always in the expected direction and showed a more nuanced picture in which differences among countries emerged. First, in line with previous research (Gattino et al., 2018; Slevec & Tiggemann, 2011), socially prescribed perfectionism was a risk factor for BSH in Italy and in Poland. However, this dimension did not play any role in fostering or protecting against BS. Second, high self-oriented perfectionism was linked to higher levels of both BSH and BS only in the Polish sample. Our data show that the association between “being perfect/successful” and physical appearance varies between countries. Previous studies linked perfectionism to body dissatisfaction, arguing that the greater need for others’ approval that perfectionism entails may be a risk factor, particularly in those cultures where the construction of the self is more interdependent (Boone et al., 2014; Bulik et al., 2003). However, the non-uniqueness of these results deserves further attention to investigate the role that both socially prescribed perfectionism and self-perfectionism can have in shaping self-objectification, especially in non-Western cultures that are still understudied.

Additionally, we investigated the effect of other sources of social influence—i.e., family, friends and one’s partner—on self-objectification. Our findings highlighted a complex pattern, which differs between countries, and not always in line with our hypotheses. In line with the literature (Arroyo & Andersen, 2016; Katz-Wise et al., 2013), the influence exerted by the family emerged as a significant predictor of body shame in all countries except Iran. However, this same variable was not associated with BS in any of the five countries. The peculiarity of the results in our Iranian sample may be due to cultural reasons. In this social context, where the hijab, which minimizes exposure in public, has a religious meaning, it is possible that parents exert less influence on the dimension of BSH than parents acting in different social contexts. Friends emerged as significant risk factors for BSH in Poland and Iran, whereas a romantic partner acted as a risk factor for BSH in Italy. Neither peer groups nor partners were found to be a significant factor for BS. It can be concluded that the role that the significant others (family, partners, friends) play in different countries is not consistent and has to be explored in more depth.

### Limitations

Our findings contribute to advancing our understanding of factors related to self-objectification examined using a research design involving people living in social contexts with social, cultural, and religious backgrounds different from those in Western or Westernized countries, usually scarcely, or not at all, included in studies of self-objectification. Another strength of this work is that the samples are men and women of different age cohorts.

Notwithstanding these strengths, our study has several limitations that may be addressed in future research. First, the cross-sectional design constrains the interpretation of causal effects. Second, the generalizability of the present research is limited because we used non-random sampling procedures. Longitudinal research with more representative groups of participants would make it possible to better understand the effects of the different variables considered and to verify any changes over time of the phenomenon under investigation. Third, the five countries included in this study were not homogeneous as there were some variations in percentage of women and mean age and this could have affected the results. An additional limitation is the inclusion of BMI in the analysis, which, being a self-reported measure, is not a fully accurate metric. Moreover, given that we included only one Middle Eastern country (i.e., Iran), the present findings would not be generalizable to other Asian countries as the hijab is legally mandated for women in Iran but not in most other Asian countries—including other Asian cultures where wearing the hijab is common but voluntary. Finally, although OBCS is one of the most widely used scales in self-objectification research, its use is problematic and other measures have recently been developed (e.g., Daniel et al., 2014; Lindner & Tantleff-Dunn, 2017; Talmon & Ginzburg, 2016) that may shed light on slightly different theoretical issues. Furthermore, in line with most cross-cultural research, the meaning of each measure used may be slightly different depending on the translation and cultural context.

### Practical Implications

The results from the current study highlight the complexity of self-objectification, where psychological and social variables play a role. There is a need to address both these dimensions, and contrast this phenomenon in a world that, because of globalization, is now increasingly small. Our findings showed the need to consider a wide range of variables related to self-objectification, within a multilevel perspective that ranges from personal to relational and cultural-contextual variables. Moreover, these findings point to the need to implement interventions that take into account political, religious, and social dimensions, going beyond just individual target dimensions such as gender and age. The sociocultural dimension must be considered both by those who set research goals and by those who intend to develop interventions aimed at preventing self-objectification and its harmful effects. Our results may guide prevention and intervention efforts in social, educational, and clinical settings from a culturally situated perspective.

### Conclusion

Taken together, the present findings suggest that self-objectification is a complex, life-long, socially constructed phenomenon. They also show that BSH and BS are two non-overlapping dimensions of self-objectification and that the antecedents underlying them differ. Because environmental antecedents play an important role in conveying ideologies that favor or protect against the experience of self-objectification (Calogero & Jost, 2011), we call for future research to examine the role of social norms and values in self-objectification, such as analyzing exposure to types of sexist ideologies in different cultural contexts. Furthermore, we hope this study will prompt practitioners, educators, and policymakers to work to make people aware of the potential risk and protective factors of self-objectification, and take action to avoid the former and to foster the latter.

## Supplementary Materials

The supplementary materials provided are two additional statistical analyses used in the research and can be accessed in the Index of Supplementary Materials below).



GattinoS.
Czepczor-BernatK.
FediA.
Brytek-MateraA.
BozaM.
LemoineJ. E.
SahlanR. N.
WilsonE.
De PiccoliN.
RolleroC.
 (2023). Supplementary materials to "Self-objectification and its biological, psychological and social predictors: A cross-cultural study in four European countries and Iran"
[Statistical analyses]. PsychOpen. 10.23668/psycharchives.12537
PMC1010305437063692

## Data Availability

Data is freely available at Supplementary Materials.
